# Development of Intelligent and Active Gelatin-Based Packaging Film Incorporating Red Onion Anthocyanins and Encapsulated Citronella Oil

**DOI:** 10.3390/foods14193320

**Published:** 2025-09-25

**Authors:** Zhaolan Yan, Kun Wang, Bingbing Xia, Jintao Wu, Hongxu Chen

**Affiliations:** 1 Faculty of Food Science and Engineering, Kunming University of Science and Technology, Kunming 650500, China; yanzhaolan107@163.com (Z.Y.); wujintao8447@163.com (J.W.); 15282529757@163.com (H.C.); 2 Sorbonne Université, Centre National de la Recherche Scientifique (CNRS), Institut des NanoSciences de Paris (INSP), la Plateforme d’Analyse par Faisceau d’ions Rapides (SAFIR), 75005 Paris, France; xiabing0626@126.com

**Keywords:** pH responsiveness, freshness monitoring, smart packaging

## Abstract

With rising living standards, consumer demand for fresher food continues to increase. Consequently, the development of multifunctional packaging materials that enable real-time freshness monitoring, delay spoilage, and ensure environmental sustainability has become a central research focus. The present study developed an antibacterial and pH-responsive smart packaging film, formulated from a κ-carrageenan/gelatin (CG) matrix. This film incorporated anthocyanins extracted from red onion skin (ROSA) and citronella essential oil encapsulated in β-cyclodextrin (OBDs) as functional additives, herein referred to as the CGR/OBDs composite film. The composite films exhibited strong pH sensitivity, ammonia responsiveness, color stability, effective barrier properties, and notable antioxidant activity (96.4% ABTS and 79.3% DPPH radical scavenging rates). The sustained release of citronella essential oil over approximately 40 h conferred excellent antibacterial performance, with inhibition rates of 94.8% against *Staphylococcus aureus* (*S. aureus*) and 91.6% against *Escherichia coli* (*E. coli*). Application in shrimp preservation further demonstrated an extended shelf life and real-time freshness monitoring through distinct colorimetric shifts. The findings highlight the potential of CGR/OBDs films as visual indicators for food freshness in intelligent packaging, offering a promising strategy to enhance food safety and reduce waste.

## 1. Introduction

Food spoilage remains a significant challenge in the food industry, contributing substantially to food waste and raising critical concerns regarding food safety [1]. The gradual and often imperceptible deterioration of fresh food quality is typically assessed through visual inspection and consumer judgment. In the absence of scientific guidance, such evaluations may result in the consumption of stale or contaminated food, posing health risks that are further amplified by inadequate packaging and the lack of effective freshness monitoring systems [2]. Among perishable food items, shrimp stands out due to its high nutritional value and pronounced vulnerability to microbial spoilage, which generates amines and causes pH fluctuations during storage [3,4]. Consequently, extensive efforts have been directed toward extending the shelf life of shrimp, reducing food waste, and enabling rapid and accurate assessment of freshness and quality.

Innovative packaging strategies, especially pH-responsive indicator systems, have emerged as promising solutions to food spoilage. These packaging systems respond to pH variations caused by food degradation and environmental changes, producing visible color shifts that serve as intuitive indicators of freshness and quality [5,6]. Recent studies have explored the use of anthocyanins from natural sources, such as purple sweet potato [7], blueberries [8], and red cabbage [9], for the development of smart pH-indicating films. Anthocyanins are water-soluble phenolic compounds whose molecular structures undergo reversible structural transformations in response to pH fluctuations, resulting in distinct color changes. In addition to pH sensitivity, anthocyanins exhibit strong antioxidant activity [10]. Wei et al. [11] reported that anthocyanins act as cross-linking agents in protein-based films, forming covalent bonds, ionic interactions, hydrophobic effects, and hydrogen bonding [12,13]. These interactions enhance film properties by improving flexibility and reducing water vapor permeability. Among potential sources of anthocyanins, red onions are widely cultivated and produce a large volume of by-products, particularly red onion skins. Given the growing emphasis on sustainability, valorization of agricultural waste such as red onion skins for anthocyanin extraction (ROSA) provides an eco-friendly pathway for functional film development [14].

pH indicator films derived from anthocyanins offer real-time assessment of food quality; however, their antibacterial efficacy remains limited or negligible. As a result, single-function films fall short of meeting the growing demand among manufacturers and consumers for multifunctional packaging solutions. Incorporation of antibacterial agents into the film matrix has been proposed as a strategy to overcome this limitation [15]. Among various options, plant-derived essential oils, such as citronella essential oil (CEO), are widely used due to their recognized safety and broad-spectrum antimicrobial activity [16,17]. Despite these advantages, the practical application of essential oils in food preservation is restricted by high volatility and chemical instability [18]. Microencapsulation provides an effective strategy for stabilizing essential oils, protecting them from heat and light degradation, and enhancing their biological activity [19,20,21]. Cyclodextrins, natural encapsulating agents with a hydrophilic exterior and a hydrophobic inner cavity, improve the solubility and stability of hydrophobic compounds, enable controlled release, and preserve bioactivity [22]. Encapsulation of the CEO within β-cyclodextrin (β-CD) to form citronella essential oil microcapsules (OBDs) offers a promising approach to enhancing oil stability and achieving a sustained antibacterial effect within pH-indicator films.

The widespread reliance on petroleum-based plastics for food packaging has raised growing environmental concerns [18]. In response, biodegradable, bio-based packaging materials have attracted increasing attention within the food industry. Gelatin (GEL), derived from thermally denatured collagen, is favored for its abundance, low cost, excellent biodegradability, and superior film-forming ability [23,24]. Carrageenan, a polysaccharide extracted from red seaweed, functions as an effective barrier against oil and oxygen while offering antioxidant protection during food storage. Among its variants, κ-carrageenan (CAR) is the most commonly used and exhibits strong interactions with proteins under defined conditions. The negatively charged anionic chains of CAR can interact with the positively charged regions of gelatin molecules, resulting in composite films with improved structural and functional integrity [25].

The present study developed a κ-carrageenan/gelatin-based composite film (CGR/OBDs) incorporating ROSA and OBDs to achieve dual functionalities of real-time freshness monitoring and antibacterial activity. Anthocyanins extracted from red onion skin waste served as a natural pH-responsive dye, enhancing structural integrity and flexibility of the films while supporting high-value utilization of agricultural by-products and reducing onion waste. Prior to application in seafood packaging, the composite films were systematically characterized for structural, physical, optical, antioxidant, and antibacterial properties. The sustained release behavior of the encapsulated essential oils was also analyzed. Additionally, the films’ colorimetric response across a range of pH values and sensitivity to ammonia vapor were investigated. The films were subsequently applied to shrimp packaging, enabling real-time freshness assessment and demonstrating the potential of multifunctional smart films for food quality monitoring applications.

## 2. Materials and Methods

### 2.1. Materials

κ-carrageenan (CAR, 15–405 sulfate, purity ≥ 90%) and citronella essential oil (CEO, purity > 99%) were purchased from Shanghai Yuanye Bio-Technology Co., Ltd. (Shanghai, China). Gelatin (GEL, molecular weight: ~60 kDa, purity ≥ 98%) was obtained from Beijing Solarbio Science & Technology Co., Ltd. (Beijing, China). β-Cyclodextrin (β-CD, molecular weight: 1134.98, purity ≥ 98%) was also procured from the same supplier. Fresh red onions were sourced from local supermarkets in Kunming.

### 2.2. Extraction of Red Onion Skins Anthocyanins

Anthocyanins were extracted following the method described by Ali et al. [26]. The extraction solvent consisted of 75% ethanol acidified with glacial acetic acid in a 6:1 ratio. Extraction efficiency was evaluated by monitoring color variations and UV–visible absorption spectra of anthocyanin solutions across a pH range of 1–12, as detailed in the Supplementary Materials.

### 2.3. Preparation and Characterization of OBDs

OBDs microcapsules were prepared using a co-precipitation technique adapted from the method reported by Chen et al. [27]. Detailed procedures for OBD synthesis are provided in the Supplementary Materials. The microstructure of OBDs was examined using a field emission scanning electron microscope (SEM, Thermo Scientific Apreo 2C, Waltham, MA, USA) operating at 5 kV, with gold sputtering applied prior to examination. The functional groups of OBDs were analyzed using a Fourier transform infrared spectrometer (FTIR, Thermo Scientific Nicolet iS5, Waltham, MA, USA) over a spectral range of 400–4000 cm^−1^, employing the KBr pellet method. Crystal form analysis was performed using an X-ray diffractometer (XRD, Bruker D8 Advance, Selb, Bavaria, Germany) at a scanning rate of 2°/min and a diffraction angle (2θ) ranging from 10° to 80°. Antioxidant and antibacterial properties of the OBDs were also evaluated using methods described in the Supplementary Materials.

### 2.4. Preparation and Characterization of Composite Films

#### 2.4.1. Preparation of Composite Films

Gelatin and κ-carrageenan powders (2 g each) were separately dissolved in 100 mL of distilled water and stirred at 70 °C until uniform dispersions were obtained, yielding 2% (*w*/*v*) solutions. The two solutions were then combined in a 1:1 ratio and stirred continuously at 45 °C. Glycerol, serving as a plasticizer, was added at 20% of the total mass of gelatin and carrageenan. Tween 80 (0.015%, *w*/*v*) was incorporated as an emulsifier. Subsequently, anthocyanins extracted from ROSA were added at 30% of the total polymer mass, and OBDs were introduced at 1%, 3%, 5%, and 7% (*w*/*w*) relative to the same basis. The resulting mixture was stirred at 45 °C until a uniform solution was obtained. Each film-forming solution was cast onto a 20 cm × 20 cm acrylic plate and dried at ambient temperature. Control films, including pure CG and CG with 30% ROSA (referred to as CGR), were prepared under identical conditions. After drying, all films were carefully peeled off and stored in desiccator at 25 °C and 50% relative humidity (RH) for 48 h to equilibrate moisture content. Film thickness was measured as 0.059 ± 0.009 mm, with no significant differences among formulations. Composite films containing varying OBDs concentrations were labeled as CGR/OBDs-1%, CGR/OBDs-3%, CGR/OBDs-5%, and CGR/OBDs-7%, respectively.

#### 2.4.2. Physicochemical Properties of Composite Films

The surface morphology of the films was examined using a field emission SEM (Thermo Scientific Apreo 2C, Waltham, MA, USA) operated at an accelerating voltage of 5 kV following gold sputtering. Functional groups were analyzed using an FTIR spectrometer (Thermo Scientific Nicolet iS5, Waltham, MA, USA) equipped with an attenuated total reflectance (ATR) accessory, over a 400–4000 cm^−1^ spectral range. Crystalline structure was characterized using XRD (Bruker D8 Advance, Selb, Bavaria, Germany) at a scanning rate of 5°/min with a 2θ range of 10° to 80°.

The thermal stability of the composite films was evaluated through thermogravimetric analysis (TG-DTG, TG 209F1 NETZSCH, Selb, Bavaria, Germany) under a nitrogen atmosphere. Samples were heated from 25 °C to 800 °C at a constant rate of 20 °C/min. The maple leaf was covered with a 4 cm × 2 cm film for photography. Optical properties were measured with a T90 UV–visible spectrophotometer (Purkinje, Beijing, China) across 200–800 nm following published procedures [28]. Film color parameters were determined using a colorimeter (NR110, 3nh Technology Co., Ltd., Shenzhen, China). Water vapor permeability (WVP) was measured following the method of Wang et al. [29], while oxygen permeability (OP) was measured based on the protocol reported by Geng et al. [30]. The mechanical properties were measured using a QLW-5E tensile machine (Qunlong Instrument Co., Ltd., Xiamen, China) under 50% RH and 25 °C following ASTM D 882–97. Film strips (80 mm × 15 mm) were prepared, with an initial grip separation of 50 mm and a crosshead speed of 50 mm/min.

#### 2.4.3. Antioxidant Activity of Composite Films

Antioxidant activity was evaluated using both DPPH and ABTS^+^ radical scavenging assays. For each test, 10 mg of film sample was mixed with 2 mL of 0.1 mM DPPH ethanol solution or ABTS^+^ solution, respectively. The procedures followed the same protocols used to evaluate the antioxidant activity of OBDs, as detailed in the Supplementary Materials.

#### 2.4.4. Antibacterial Activity and Mechanism of Composite Films

The antibacterial activity of the composite films was evaluated using the plate counting method described by Li et al. [31], targeting Gram-negative *Escherichia coli* (*E. coli*, ATCC25922) and Gram-positive *Staphylococcus aureus* (*S. aureus*, ATCC6538). Petri dishes with a diameter of 9 cm and a height of 1.5 cm were used. Detailed procedures are provided in the Supplementary Materials. SEM (SU8010, Hitachi High-Technologies, Tokyo, Japan) was employed to investigate the antibacterial mechanism against both bacterial strains. Experimental groups included bacterial cultures treated with CG, CGR, and CGR/OBDs-5% films [32], while untreated cultures served as controls. Bacteria were grown in LB medium, centrifuged at 1500–3000 rpm for 5–10 min, and washed twice with PBS. The pellets were fixed in 2.5% glutaraldehyde at 4 °C overnight. Prior to SEM imaging, samples were vacuum-dried and gold sputter-coated.

#### 2.4.5. Biosafety Testing, Degradation Testing and Release of CEO of Composite Films

Blood compatibility testing was conducted according to the protocol established by Li et al. [33]. The overall migration (OM) test followed the procedure reported by Liu et al. [32]. Biodegradability was evaluated under natural outdoor conditions. Film samples (4 cm × 4 cm) were buried in soil at a depth of 15 cm in Kunming, China (temperature 15–26 °C, relative humidity 50–70%, August). Films were inspected every 4 days, and visual changes were recorded. Commercial polyethylene (PE) film was used as a reference.

The release behavior of citronella essential oil from the composite films was evaluated using food-simulating liquids under various temperatures based on the method reported by Lian et al. [34]. Simulants included 3% acetic acid (acidic food), 50% ethanol (alcoholic food), and 95% ethanol (fatty food). Samples were incubated at 4 °C, 25 °C, and 37 °C for up to 130 h. At predetermined time points (0, 1, 3, 6, and 12 h), aliquots were collected for UV–visible spectrophotometric analysis, and the withdrawn volume was replenished to maintain 10 mL. Full-wavelength scans identified 214 nm as the maximum absorption wavelength of CEO.

#### 2.4.6. pH-Responsive and Color Stability of Composite Films

pH sensitivity and color stability of the films were assessed following the procedures described by Hu et al. [35]. To evaluate color stability, film samples were stored under varying light and temperature conditions for 28 days. Changes in color (Δ*E*) were measured at 7-day intervals using a handheld colorimeter. Storage environments included 25 °C under light, 25 °C in darkness, 4 °C under light, and 4 °C in darkness. An incandescent lamp was used as the light source during the experiment, with a color temperature of 2800 K.

#### 2.4.7. Ammonia-Sensitive Performance of Composite Films

Ammonia sensitivity was examined following the method of Tang et al. [36]. Film samples (2 cm × 2 cm) were suspended 3 cm above 20 mL of concentrated ammonia solution for 10 min. Color changes were recorded at 2 min intervals using a colorimeter, and photographs were taken throughout the exposure period.

### 2.5. Application of Composite Films

Fresh shrimp were obtained from a local market in Kunming, China, and stored at 4 °C for five days to investigate the films’ colorimetric indication of freshness. Shrimp samples of similar weight (20 ± 2 g) were placed in Petri dishes (13 cm in diameter and 2.5 cm in height) and assigned to four packaging conditions: unwrapped, wrapped with polyethylene (PE) cling film, covered with CGR film, and covered with CGR/OBDs-5% film (film dimensions: 20 cm × 20 cm). During storage, shrimp quality was assessed at predetermined intervals. Measured parameters included pH, total volatile base nitrogen (TVB-N, mg/100 g), total viable count (TVC, log CFU/g), and the a*, b*, and Δ*E* values of the films. pH was measured according to the GB 5009.237-2016 standard. TVB-N was determined by the semi-micro Kjeldahl method in accordance with GB/T 5009.44-2003, and TVC was assessed following the GB 4789.2-2022 protocol.

### 2.6. Statistical Analysis

All performance tests were conducted in triplicate, and the results are presented as mean ± standard deviation. Statistical significance among the data was assessed using a one-way analysis of variance (ANOVA) and Duncan’s multiple range test, with a significance threshold set at *p* < 0.05.

## 3. Results and Discussion

### 3.1. Characterization of OBDs

**Figure 1:**
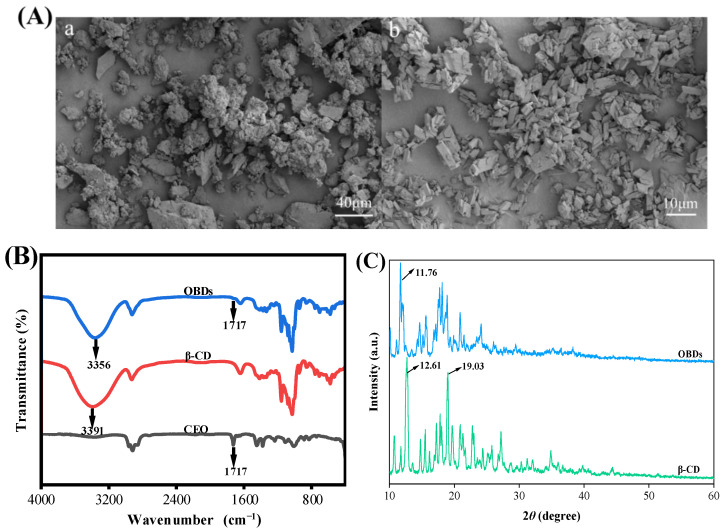

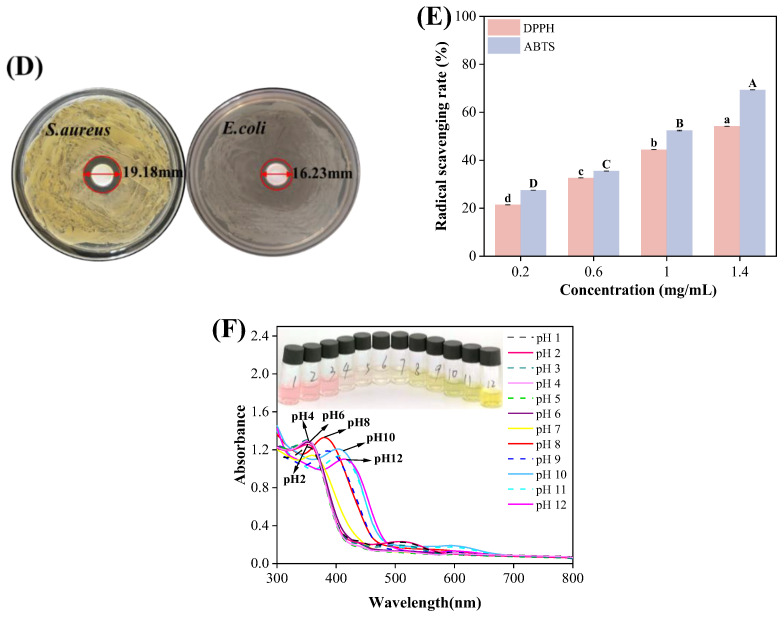
(**A**) SEM images of (**a**) β-CD and (**b**) OBDs. (**B**) FTIR spectrum of CEO, β-CD and OBDs. (**C**) XRD patterns of β-CD and OBDs. (**D**) The antibacterial activity of OBDs against *S. aureus* and *E. coli*. (**E**) The antioxidant activity of OBDs. (**F**) Color changes (inset) and UV-visible spectra of anthocyanin solutions in different pH solutions (pH 1–12). Different letters (a–d, A–D) indicated significant differences within groups (*p* < 0.05).

The encapsulation efficiency of OBDs was calculated as 73.78%, following the method of Chen et al. [27]. The SEM images of β-CD and OBDs are presented in Figure 1A. The microstructure of β-CD appeared irregular, with numerous aggregated cuboid-like structures. In contrast, the OBDs exhibited a predominantly rhombohedral morphology with sharp edges and corners, consistent with previous findings reported by Shi et al. [37]. The FTIR spectra of CEO, β-CD and OBDs are presented in Figure 1B. The CEO exhibited a characteristic C=O stretching vibration at 1717 cm^−1^, corresponding to the carbonyl groups of aldehydes or esters—key chemical constituents of the oil. In the FTIR spectrum of OBDs, a similar carbonyl absorption was observed at 1717 cm^−1^, while this peak was absent in the spectrum of β-CD, indicating the presence of CEO in the OBDs. The relatively weak intensity of the 1717 cm^−1^ peak in the OBDs suggests effective encapsulation of CEO within the β-CD matrix, likely due to the shielding effect provided by the β-CD cavity [38]. Additionally, a red shift in the O–H stretching vibration from 3391 cm^−1^ in β-CD to 3356 cm^−1^ in OBDs indicated enhanced hydrogen bonding interactions between CEO and β-CD. These interactions contributed to the formation of a more compact and stable microcapsule structure [39]. XRD patterns of β-CD and OBDs are shown in Figure 1C. β-CD exhibited sharp diffraction peaks at 2θ = 12.61° and 19.03°, characteristic of its cage-like crystalline structure. In contrast, OBDs displayed a principal peak at 2θ = 11.76°, along with a broad range of channel-like peaks between 13° and 22°. These changes in peak position, shape, and intensity reflect significant structural rearrangements upon CEO encapsulation, suggesting a transition of β-CD from its native cage-like lattice to a layered arrangement following inclusion complex formation [40]. Collectively, these results confirm the successful preparation of OBDs through the incorporation of CEO into the β-CD matrix.

### 3.2. Antioxidant and Antibacterial Activity of OBDs

The antibacterial activity of OBDs was evaluated using the agar diffusion method. As illustrated in Figure 1D, OBDs exhibited antibacterial effects on both *S. aureus* and *E. coli*, with inhibition zone diameters of 19.18 mm and 16.23 mm, respectively. Subsequently, the antioxidant activity of OBDs was assessed, as depicted in Figure 1E. Notably, as the concentration of OBDs increased, the DPPH and ABTS scavenging rates also significantly increased, with the highest ABTS free radical scavenging rate reaching 69.4%. The pronounced antioxidant activity observed in the OBDs may be attributed to the presence of key bioactive compounds, such as citronellal, geraniol, and citronellol, which exhibit strong free radical-scavenging capabilities, thereby delaying lipid oxidation processes. In terms of antibacterial performance, the OBDs demonstrated notable inhibitory effects against both Gram-positive bacteria (*S. aureus*) and Gram-negative bacteria (*E. coli*). This broad-spectrum activity is likely due to the ability of citronella oil components to compromise the integrity of bacterial cell membranes, resulting in the leakage of intracellular contents and subsequent cell death [41,42].

### 3.3. UV-Vis Spectroscopy of Anthocyanins

Color transitions and UV–visible spectra of ROSA solutions across pH 1–12 are shown in Figure 1F. As pH increased, the solution exhibited distinct color changes, transitioning from light pink at pH 1 to yellow-green at pH 12. These variations were attributed to pH-induced structural transformations of anthocyanin molecules. At pH values below 3, anthocyanins predominantly existed as flavylium cations, producing a characteristic light pink color. Between pH 3 and 6, progressive deprotonation led to the formation of carbinol pseudobases, resulting in a gradual loss of color intensity. At alkaline conditions above pH 7, anthocyanins converted into chalcone structures, yielding a yellow-green solution [43]. The observed behavior confirmed that ROSA anthocyanins were highly sensitive to pH variations. Changes in the UV–visible spectra closely mirrored the corresponding color transitions of the solution. Both the intensity and wavelength of the maximum absorption peak varied with pH. Under strongly acidic conditions (pH < 4), a prominent absorption peak appeared near 350 nm, with peak intensity gradually decreasing as pH increased. These results demonstrate the strong pH sensitivity of ROSA anthocyanins and support their application as natural indicators in smart packaging films for real-time food freshness monitoring.

### 3.4. Characterization of Films

**Figure 2:**
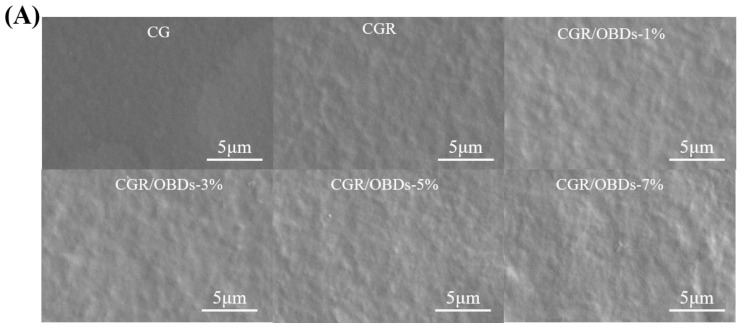

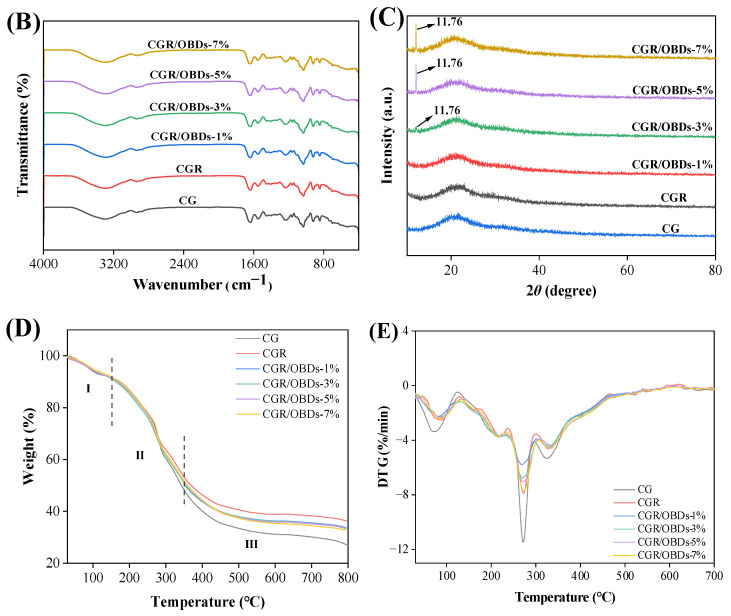
(**A**) SEM, (**B**) FTIR, (**C**) XRD patterns, (**D**) TG and (**E**) DTG curves of different composite films.

The morphology of the composite films was assessed through SEM, as shown in Figure 2A. The CG film exhibited a smooth and uniform surface free of pores or cracks. After the incorporation of ROSA, the surface remained generally homogeneous with slight wrinkling, suggesting good compatibility among the film components. The subsequent addition of essential oil microcapsules into the CGR matrix produced films with relatively dense and consistent surfaces at 1%, 3%, and 5% OBDs concentrations. However, at 7% OBDs, noticeable aggregation occurred, leading to surface roughness and heterogeneity. These morphological changes indicated reduced compatibility between the biopolymer matrix and the encapsulated essential oil [44,45]. Figure 2B presents the FTIR spectra of various composite films within the range of 4000–400 cm^−1^. Key functional groups included O–H (from ROSA, OBDs, GEL, and CAR) and N–H (from GEL). Vibrational shifts in these bands may reflect interactions among film constituents. The relatively low OBDs content compared with the high proportion of CG likely masked the characteristic peaks of OBDs, as similarly reported in prior studies. For instance, the addition of thyme essential oil to soy protein isolate showed a comparable masking effect [46]. The crystal formation among GEL, CAR, ROSA, and OBDs were analyzed using X-ray diffraction, as illustrated in Figure 2C. The CG film displayed a broad peak centered around 2θ = 20°, suggesting a predominantly amorphous structure. Composite films containing different additives also displayed broad peaks, consistent with the amorphous nature of the biopolymer matrix. Incorporation of ROSA did not induce notable changes in the XRD patterns, suggesting that anthocyanins were uniformly distributed within the matrix and showed good compatibility with CG [47,48]. In contrast, the composite film containing 3%, 5%, and 7% OBDs displayed a peak at 2θ = 11.76°, along with several new crystalline peaks. These features correspond to those observed in the XRD pattern of pure OBDs and reflect the crystalline nature of the encapsulated microcapsules. Despite these additional peaks, the overall XRD profiles remained largely amorphous, indicating that ROSA and OBDs did not substantially disrupt the physical state of the biopolymer matrix [49].

### 3.5. Thermal Stability of Films

Thermogravimetric analysis was employed to evaluate the thermal stability of the films by monitoring mass loss during controlled heating. As illustrated in Figure 2D, all films exhibited three distinct stages of mass degradation. The first stage, between 30 °C and 150 °C, corresponded to the evaporation of adsorbed moisture, crystal water, and residual solvents. The second stage, from 150 °C to 350 °C, involved substantial weight loss caused by volatilization of residual glycerol and thermal decomposition of GEL, CAR, anthocyanins, and β-CD. Degradation of β-CD was accompanied by the release of citronella essential oil [50]. The maximum weight loss rate (Tmax) occurred near 271 °C, corresponding to the breakdown of the gelatin protein chains and the disruption of the polysaccharide structure in CAR [51]. The third stage of decomposition, spanning 350 °C to 800 °C, reflected further degradation, fracture, and potential carbonization of the film matrix [52]. Residual weights at 800 °C were 26.83% for CG and 36.27% for CGR films. For CGR/OBDs films, the residual masses were 30.72%, 33.25%, 33.50%, and 32.70% at OBDs concentrations of 1%, 3%, 5%, and 7%, respectively. In the DTG curve, the Tmax of CGR and CGR/OBDs values for the initial and third stages in CGR and CGR/OBDs films shifted to higher temperatures compared to the CG film, while the peak intensities of Tmax in all three stages decreased significantly. These results suggest that the addition of ROSA improved the thermal stability of the CG matrix, likely due to the formation of cross-links between anthocyanins and the biopolymer components, resulting in a denser film structure. Incorporation of OBDs increased the total decomposition mass relative to CGR, likely due to essential oil dispersion, while reducing the overall decomposition rate, indicating a stabilizing effect of OBDs on the film matrix. A similar enhancement in thermal stability following essential oil microencapsulation was reported by Akrami et al. [53]. In contrast, other studies have shown that the inclusion of anthocyanins from black wolfberry and bayberry pomace slightly reduced the thermal stability of pullulan/PVA-based films [54,55].

### 3.6. Optical Properties of Films

The color and transparency of packaging films play a critical role in determining visual appeal and consumer acceptance. Color parameters of the films are presented in Table S1. Notably, the incorporation of ROSA into the CG film enhanced a* (redness) and b* (yellowness) values, indicating a slight yellow tint. This coloration was attributed to the light absorption properties of anthocyanins and phenolic compounds present in the extract [56]. As OBDs content increased, the a* (redness) value decreased while the b* (yellowness) value increased. A corresponding increase in the total color difference (Δ*E*) was also observed, indicating that the CGR/OBDs films developed a more intense color. Such coloration is advantageous for protecting packaged food from both visible and ultraviolet (UV) light exposure. As illustrated in Figure S1A, all films exhibited a smooth and uniform appearance. The UV-visible transmittance spectrum of the composite film, spanning 200–800 nm, is depicted in Figure S1B. The addition of ROSA and OBDs significantly reduced transmittance in the 200–400 nm range, demonstrating excellent UV-blocking performance. This effect was primarily attributed to the conjugated double-bond structures of anthocyanins, which enable strong electronic transitions in the UV region. These transitions facilitate the absorption of UV light and its conversion to thermal energy, thereby limiting UV transmission [44]. In addition, CEO possesses intrinsic UV-absorbing properties that were further enhanced when encapsulated in β-CD, due to improved dispersion and stability [45]. Consequently, compared to the CG film, composite films containing ROSA and OBDs exhibited superior resistance to UV–visible light, a property essential for inhibiting lipid oxidation and preserving the sensory quality of packaged foods.

### 3.7. Barrier Properties and Mechanical Properties

**Figure 3:**
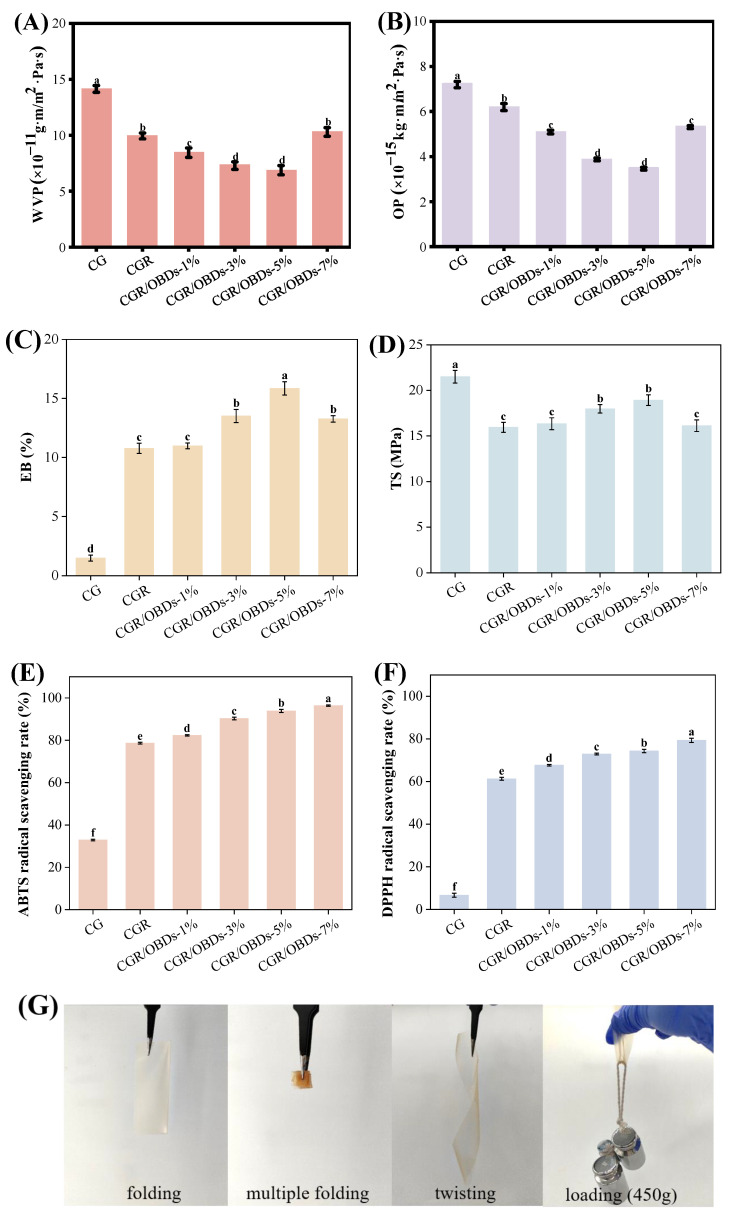
Barrier properties (**A**) WVP and (**B**) OP. Mechanical properties (**C**) EB and (**D**) TS. And antioxidant capacities (**E**) ABTS and (**F**) DPPH radical scavenging rates of various composite films. (**G**) Photographs of CGR/OBDs-5% composite film under folding, multiple folding, twisting and loading with 450 g. Different lowercase letters (a–f) indicated significant differences within groups (*p* < 0.05).

Packaging films with suitable barrier properties are essential for reducing moisture and gas exchange between food and the external environment, thereby preventing spoilage and extending shelf life. The WVP and OP values of CG films with ROSA and varying OBDs concentrations are shown in Figure 3A,B. Incorporation of ROSA significantly reduced both WVP and OP compared with CG, likely due to cross-linking interactions between anthocyanins and the biopolymer matrix that produced a denser structure. Among the CGR/OBDs films, those with 1%, 3%, and 5% OBDs demonstrated further reductions in WVP, while the film containing 7% OBDs showed a notable increase. The presence of OBDs enhanced film thickness and compactness, effectively impeding moisture and gas diffusion. This improvement was attributed to the filling effect of the microcapsules, which reinforced the film matrix and enhanced structural integrity. However, excessive OBDs content disrupted the film network, creating loose pores within the cross-linked matrix. These microstructural defects facilitated oxygen and water vapor transmission, leading to increased WVP and OP values at higher OBDs concentrations [57,58].

Mechanical properties are critical indicators of the functional performance of food packaging materials. As shown in Figure 3C,D, the elongation at break (EB) and tensile strength (TS) of the CG film were recorded at 1.47% and 21.51 MPa, respectively. Incorporation of ROSA significantly increased EB but reduced TS. The increase in EB was attributed to the polyphenolic structure of anthocyanins, which promoted cross-linking with gelatin and κ-carrageenan and enhanced film flexibility. The reduction in TS was likely caused by the disruption of the polymer network by phenolic compounds, which increased elasticity but decreased rigidity [59]. Subsequent incorporation of OBDs into the CGR matrix resulted in concentration-dependent changes in mechanical properties. With increasing OBDs content, both EB and TS initially increased, reaching optimal values at 3% and 5% concentrations before declining at higher levels. A similar trend was reported by Zhang et al. [50] in films containing oregano essential oil-loaded β-cyclodextrin and anthocyanins. The enhanced EB at moderate OBDs concentrations was attributed to the plasticizing effect of the microcapsules, which increased molecular mobility and ductility [60]. The concurrent increase in TS was attributed to the filling effect of OBDs, which reinforced the internal structure and improved cohesion. However, at 7% OBDs, both EB and TS declined, likely due to microcapsule aggregation that disrupted uniformity, weakened the interactions among CAR, GEL, and ROSA, and compromised overall structural integrity [61]. Furthermore, the deformation capabilities of films at under practical packaging conditions (4 °C, 75% ± 5% humidity) are shown in Figure 3G. The CGR/OBDs-5% film withstood repeated folding, twisting, and a 450 g load without rupture, demonstrating excellent strength and flexibility for packaging applications.

### 3.8. Antioxidant Activity of Films

The generation of free radicals contributes to the oxidation of lipids and proteins, leading to the deterioration of food quality. Therefore, the antioxidant properties of packaging materials are critical for prolonging the shelf life of food products. The antioxidant activity of the films was evaluated using DPPH and ABTS radical scavenging assays, as depicted in Figure 3E,F. The CG composite film exhibited ABTS and DPPH scavenging rates of 32.91% and 6.68%, respectively, indicating limited intrinsic antioxidant capacity and a relatively weak ability to donate hydrogen for neutralizing free radicals. The incorporation of ROSA statistically enhanced antioxidant performance, increasing ABTS and DPPH scavenging rates to 78.69% and 61.26%, respectively. This improvement was attributed to the high free radical scavenging capacity of anthocyanins, which contributed substantially to the antioxidant activity of the film [62]. Further enhancement was observed upon the addition of OBDs. At a concentration of 5%, ABTS and DPPH scavenging rates reached 93.89% and 74.31%, respectively. When the OBDs concentration was increased to 7%, the scavenging rates further improved to 96.41% for ABTS and 79.30% for DPPH. These results demonstrate that the synergistic incorporation of ROSA and OBDs significantly boosted the antioxidant potential of the composite films, supporting their suitability for use in active food packaging aimed at preventing oxidative spoilage.

### 3.9. Antibacterial Activity of Films

**Figure 4:**
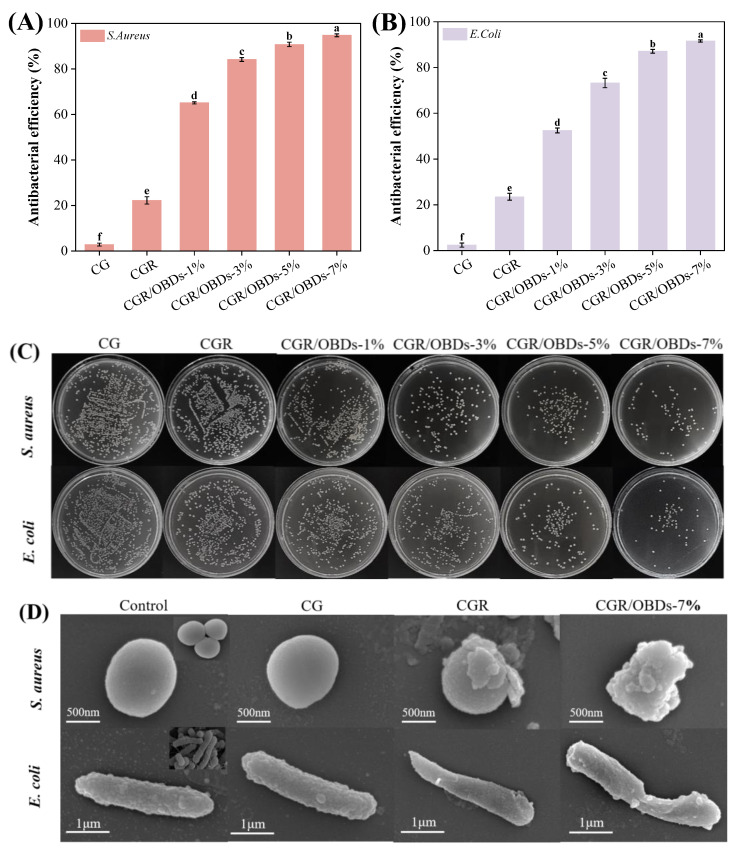
Antibacterial rates of different composite films against (**A**) *S. aureus* and (**B**) *E. coli.* (**C**) Growth of two bacteria on different films, and (**D**) SEM images of bacterial cells treated with different composite films. Different lowercase letters (a–f) indicated significant differences within groups (*p* < 0.05).

*S. aureus* and *E. coli* represent two of the most common Gram-positive and Gram-negative bacteria responsible for food spoilage. Antibacterial activity of the films against these pathogens is shown in Figure 4A–C. The CGR film exhibited a moderate antibacterial rate of approximately 22%, which represented a notable improvement over the CG film but remained limited in efficacy. In contrast, the incorporation of OBDs resulted in a concentration-dependent enhancement of antibacterial activity. At a 7% OBDs loading, inhibition rates against *S. aureus* and *E. coli* reached 94.80% and 91.60%, respectively, indicating excellent antimicrobial performance in OBDs-enriched films. The morphological effects of film treatments on bacterial cells were examined using SEM (Figure 4D). Untreated *S. aureus* and *E. coli* cells exhibited normal morphology, with intact cell walls, smooth surfaces, and typical spherical or rod-like shapes, respectively [63]. Minimal morphological disruption was observed in bacteria exposed to CGR film. In contrast, cells treated with CGR/OBDs-7% film exhibited severe structural damage, including cell membrane deformation, membrane rupture, and leakage of intracellular components, consistent with cell apoptosis. These alterations were attributed to the synergistic effects of ROSA and OBDs. Phenolic compounds in ROSA are capable of penetrating bacterial membranes and binding to intracellular proteins, thereby inactivating enzymes and inhibiting growth [64]. As indicated in Figure 1B, CEO contains carbonyl groups (C=O), which can disrupt lipids and cell walls, forming permeable pores that promote cytoplasmic leakage and ultimately result in bacterial death.

### 3.10. Biosafety and Degradability of Films

Given the intended application of these films in smart food packaging, in vitro biocompatibility was also assessed (Figure S2A). Water and PBS buffer served as positive and negative controls, respectively. After incubation with 4% rabbit red blood cells for 1 h, all composite films exhibited hemolysis rates below 5%, in compliance with ASTM standards. These results demonstrated favorable biocompatibility, supporting the potential of the films for direct contact with food products.

Overall migration (OM) values were determined in different food simulants, including distilled water, 50% *v*/*v* ethanol, 3% *w*/*v* acetic acid, and n-heptane, representing aqueous, alcoholic, acidic, and fatty environments (Figure S2B). The lowest OM values were recorded in distilled water, while the highest were observed in n-heptane. The elevated migration in n-heptane was attributed to its non-polar characteristics, which promoted swelling of the films, increased structural looseness, and facilitated dissolution of film components [65]. The incorporation of anthocyanins and OBDs significantly reduced migration rates, and all OM values remained below the regulatory threshold of 10 mg/dm^2^, confirming the films’ compliance with food packaging safety requirements [66].

Biodegradability was further examined under natural soil conditions using PE film as a reference (Figure S2C). The prepared films exhibited approximately 50% degradation within 8 days, suggesting the potential for complete decomposition over a longer period. Films containing anthocyanins and OBDs degraded more slowly than the CG film, likely due to enhanced water resistance and antimicrobial activity that limited both moisture penetration and microbial colonization [67]. Conversely, the PE film exhibited no significant signs of degradation during the testing period. These results underscore the eco-friendly and biodegradable nature of the CGR/OBDs films, highlighting their potential as a sustainable alternative for food packaging applications.

### 3.11. Release of CEO

Due to the inherent volatility of essential oils and their susceptibility to accelerated evaporation under heat, effective encapsulation is essential to ensure stability and sustained release. The release behavior of the CEO from smart films was evaluated under simulated acidic, alcoholic, and fatty food environments. As illustrated in Figure S3A–C, the release rate of CEO from CGR/CEO-5% was lower than that from the CGR/OBDs-5% film. The difference was attributed to CEO loss during the fabrication of CGR/CEO-5%, as heating at 45 °C and exposure to air accelerated volatilization, resulting in a lower retained concentration in the film matrix. Encapsulation of CEO in β-CD extended the release duration to approximately 40 h, primarily due to host–guest complexation between β-CD and CEO components driven by hydrophobic interactions and hydrogen bonding. Variations in release rates across food simulants were linked to the chemical properties of the media. In 50% ethanol, moderate polarity facilitated partial dissociation of inclusion complexes, resulting in higher release. In 95% ethanol, the strong organic solvent promoted the extraction of CEO components, while the enhanced solubility of some volatiles moderated the net release. In 3% acetic acid, the acidic environment strengthened hydrogen bonding between β-CD and protonated CEO groups, slowing diffusion and reducing volatility. The overall release behavior reflected a dynamic equilibrium process rather than simple dissolution. Lower polarity environments displayed stronger competition for the β-CD cavity and higher affinity for guest molecules, thereby accelerating release [68,69]. Temperature also had a pronounced effect on release kinetics, as shown in Figure S3D–F. Across all three solvents, the release rate of CEO peaked at 37 °C, where increased molecular mobility enhanced diffusion from the β-CD cavity. At lower temperatures, reduced kinetic energy limited molecular motion and delayed release [70,71]. These results demonstrated that β-CD encapsulation is critical for achieving controlled and sustained CEO release under diverse food storage conditions.

### 3.12. pH-Responsive and Color Stability of Films

Table 1 shows the color changes and corresponding color parameters of the CGR and CGR/OBDs-5% films across a range of pH values. All samples immersed in different buffer solutions exhibited distinct and visually distinguishable color changes, indicating that the incorporation of OBDs had minimal effect on the pH-sensitive color response of the films. Both films appeared light pink at pH 2–6, nearly colorless at pH 7, and yellow-green at pH 8–12. Significant alterations were observed in the *L* *, a*, and b* parameters (*p* < 0.05), confirming that film color responded strongly to pH variations. The variation in color difference (Δ*E*) values is recognized as the most sensitive and effective metric for detecting perceptible color changes in colorimetric sensing applications. Prior research has established that Δ*E* values greater than 5 are perceptible to the human eye [54]. In this study, even within the narrow pH range of 6 to 7, where the films transitioned from light pink to nearly colorless, the Δ*E* value exceeded 5 (for instance, the CGR/OBDs-5% film exhibited a Δ*E* difference of 5.70 between pH 6 and 7), ensuring a visually distinguishable change. All films demonstrated Δ*E* value differences greater than 5 across the entire pH spectrum, demonstrating high sensitivity and reliability for spoilage detection. In practical food packaging applications, film color changes are influenced not only by pH variations in the food matrix but also by volatile amine compounds such as ammonia, which are generated during spoilage and diffuse into the films’ microenvironment, contributing to the observed response [72,73].

**Figure 5:**
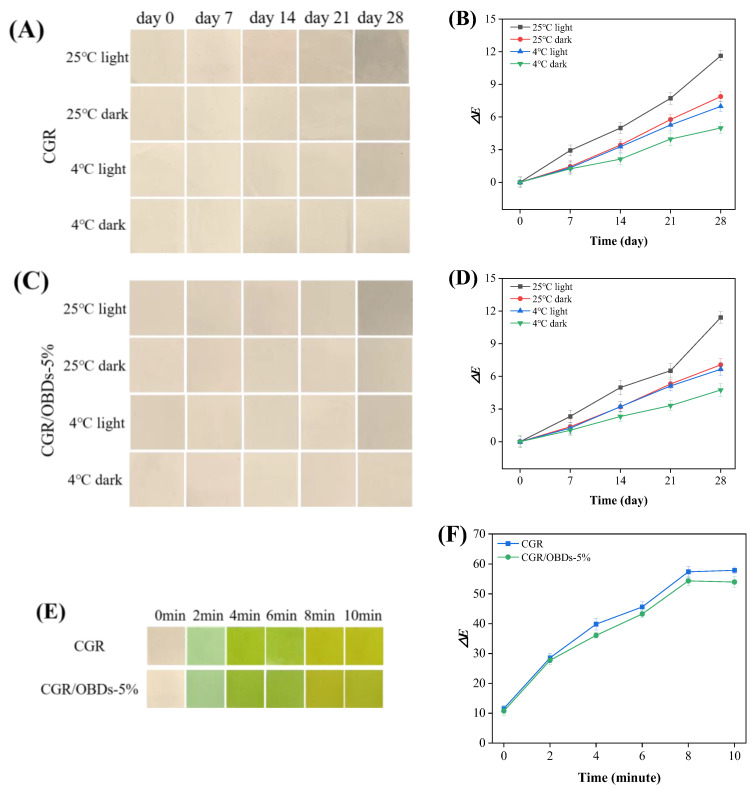
Color stability evaluation of films under different storage conditions: (**A**) The photographs taken over a 28-day period and (**B**) the corresponding changes in the Δ*E* value of the CGR film. (**C**) The 28-day photographs and (**D**) the changes in the Δ*E* value of the CGR/OBDs-5% film. Determination of ammonia sensitivity of various composite films: (**E**) photographs of films’ color changes and (**F**) the changes in the Δ*E* value.

Many natural pigments, including anthocyanins, are photosensitive, and light exposure can accelerate their degradation [74,75]. Therefore, color stability is essential for the functional reliability of smart indicating films. Long-term performance was evaluated by storing films under different light and temperature conditions, with Δ*E* values monitored over 28 days (Figure 5A–D). Under light at 25 °C, both films exhibited a progressive increase in Δ*E*, with noticeable color degradation occurring after 14 days. Films stored in darkness at 25 °C or under light at 4 °C maintained color stability for up to 21 days. The highest stability was observed in films stored in darkness at 4 °C, where Δ*E* remained below 5 throughout the 28-day period. These findings highlighted the sensitivity of anthocyanins to environmental factors such as temperature, light, and pH, consistent with observations reported by Hu et al. [35]. Overall, CGR and CGR/OBDs-5% films maintained acceptable color stability (Δ*E* < 5) for up to 14 days under all tested conditions. Considering that shrimp typically spoil within 5 days at 4 °C, the indicator films provided sufficient stability for monitoring freshness throughout the product’s shelf life. Additionally, UV radiation also influenced film stability, as shown in Figure S4. Following 4 h of exposure, the color of the CGR film gradually faded, whereas the OBDs-incorporated film displayed less pronounced changes. The color fading may be attributed to UV-induced oxidation of phenolic hydroxyl groups [76], while the presence of OBDs appeared to enhance resistance against such degradation.

### 3.13. Ammonia-Sensitive Performance

Figure 5E illustrates the colorimetric response of the CGR and CGR/OBDs-5% films when exposed to volatile ammonia. Both films exhibited a clear color transition from light green to yellow-green, indicating sensitivity to ammonia vapors. The total color difference (Δ*E*) of the CGR/OBDs-5% film increased markedly from 10.73 to over 50 within 10 min (Figure 5F), confirming that the color changes were readily visible to the naked eye. The Δ*E* of the CGR film rose from 11.58 to 57.91, significantly exceeding that of the CGR/OBDs-5% film. However, the incorporation of 5% OBDs did not significantly alter the extent or visual clarity of the color change. These findings demonstrate that CGR/OBDs-5% films possess a rapid and pronounced colorimetric response to volatile ammonia, highlighting their suitability for smart food packaging applications [77,78].

### 3.14. Application of Films

**Figure 6:**
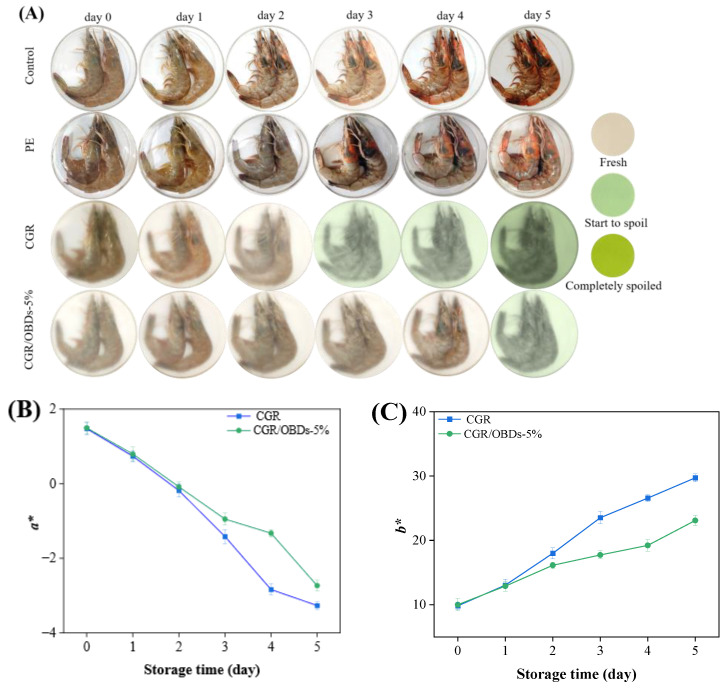

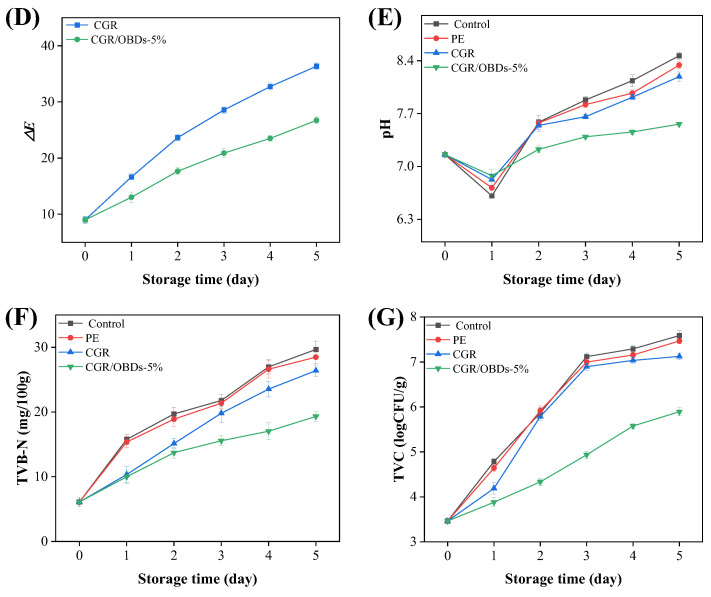
(**A**) Photographs of the shrimps preserved and monitored with different films at 4 °C. The changes in color parameters (a* (**B**), b* (**C**) and Δ*E* (**D**)) of the indicator films during the storage. The alterations in pH (**E**), TVB-N (**F**), and TVC (**G**) of shrimps during the storage.

To assess the practical performance of the smart packaging material, the CGR/OBDs-5% composite film, identified as the optimal formulation, was applied for shrimp preservation at 4 °C. Three key indicators were monitored to assess shrimp spoilage: pH, TVB-N, and TVC, along with the color difference (Δ*E*) of the smart packaging films. As illustrated in Figure 6A, on day 0, all shrimp samples across the four packaging groups appeared fresh, with translucent shells, gray coloration, and a plump, glossy texture. By day 2, shrimp in both the control and PE groups displayed early spoilage signs, including distinct red and black spots, indicating that air exposure or conventional plastic packaging could not effectively prevent deterioration. By day 3, these defects became more pronounced in the control and PE groups. Although shrimp in the CGR group showed slightly better quality, visible spoilage was still evident, accompanied by a film color shift from yellow to light green, suggesting limited antimicrobial protection. In contrast, shrimp packaged with CGR/OBDs-5% retained a fresh appearance, exhibiting intact color and plump morphology with minimal visible spoilage. These findings demonstrate that OBDs incorporation enhanced the antimicrobial and freshness-preserving performance of the films. By day 5, severe spoilage was observed in the control and PE groups, with shrimp surfaces covered by extensive black spots. In the CGR group, the film turned dark green and deterioration became more apparent. Notably, shrimp packaged with CGR/OBDs-5% still retained relatively normal color and morphology, with only mild quality decline. Spoilage indicators such as surface blackening and turbidity were substantially less pronounced compared with other groups. These results provide strong evidence that the CGR/OBDs-5% film effectively extends shrimp shelf life and maintains freshness during refrigerated storage.

Rapid shrimp spoilage results in the release of substantial amounts of alkaline volatile compounds, including ammonia and amines. These gases interact with the film, inducing a rapid color transition from yellow to green. The speed and intensity of the color change correlate directly with the degree of spoilage. The images clearly illustrate the real-time indicator function of the film, enabling consumers or distributors to intuitively assess shrimp freshness by observing the color shift without opening the package. A darker green appearance corresponds to a higher level of spoilage, as further confirmed by the Δ*E* changes shown in Figure 6B–D.

As shown in Figure 6E, shrimp pH decreased sharply on day 1, likely due to the activity of acid-producing bacteria, and rebounded on day 2 as volatile compounds such as ammonia and other amines, generated from protein degradation, were reabsorbed. The subsequent increase from near-neutral levels (~pH 7.0) in fresh shrimp to the spoilage threshold (>7.7) aligned with the films’ most sensitive response range to alkaline substances (pH 7–12), thereby inducing noticeable color changes (Δ*E* > 5) [79].

TVB-N (primarily composed of ammonia and amine compounds) serves as a key indicator of spoilage in meat and seafood. As shown in Figure 6F, the CGR/OBDs-5% film significantly delayed shrimp spoilage compared with the Control and PE film groups, extending the time to exceed the threshold value of 20 mg/100 g by approximately three days [80,81]. This preservation effect is mainly attributed to the antimicrobial properties of OBDs. As illustrated in Figure 6G, the TVC of shrimp packaged with the CGR/OBDs-5% film remained below the acceptable limit (6 log CFU/g) throughout the storage period [82].

Nonlinear regression analysis further revealed strong correlations between the Δ*E* values of the indicator film and the TVB-N, TVC, and pH values of the shrimp (Figure S5). Most importantly, the strongest correlation was observed between Δ*E* and TVB-N, suggesting that film color change was influenced not only by pH variation in shrimp flesh but also by volatile alkaline nitrogenous compounds generated during spoilage. These findings demonstrate that Δ*E* serves as a reliable and quantifiable proxy for spoilage degree, and that the color change in the film accurately reflects shrimp freshness, enabling real-time and non-destructive monitoring of seafood quality.

In summary, a comprehensive evaluation of TVB-N, TVC, pH, and colorimetric response at 4 °C demonstrated that the CGR/OBDs-5% film extended shrimp shelf life by approximately three days compared with the Control and PE groups. The developed smart composite film enhanced preservation performance and provided a visual indication of spoilage, underscoring its strong potential for intelligent food packaging applications.

## 4. Conclusions

This study successfully developed a multifunctional smart packaging film by incorporating anthocyanins extracted from ROSA and microencapsulated OBDs into a CG matrix. SEM and FTIR analyses confirmed good compatibility between ROSA, OBDs, and the CG matrix. Among the formulations, the CGR/OBDs-5% composite film demonstrated superior performance, exhibiting strong antibacterial and antioxidant activities, good blood compatibility, effective UV-blocking ability, and robust structural integrity. The film significantly inhibited microbial growth, contributing to delayed food spoilage, while maintaining biocompatibility suitable for food contact applications. Notably, the film displayed marked pH responsiveness, high ammonia sensitivity, and stable colorimetric properties for up to 14 days. In shrimp preservation experiments, the Δ*E* values of the CGR/OBDs-5% film correlated strongly with shrimp quality indicators (pH, TVB-N, and TVC), allowing the accurate visual assessment of freshness. Furthermore, the film extended the shrimp’s shelf life by approximately three days compared to conventional packaging, underscoring its practical applicability. Despite these promising results, comprehensive cost analysis and scale-up production trials are required for industrial translation. Future work will focus on optimizing film formulations for large-scale manufacture, assessing energy consumption, and conducting detailed techno-economic evaluations in line with industrial standards.

## Figures and Tables

**Table 1:** The color response of the films at pH 2–12.

Film		pH 2	pH 4	pH 6	pH 7	pH 8	pH 10	pH 12
CGR	Color	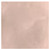	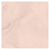	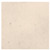	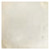	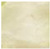	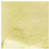	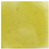
	L*	83.94 ± 0.08 ^e^	85.41 ± 0.05 ^b^	87.54 ± 0.18 ^a^	85.17 ± 0.38 ^b,c^	84.51 ± 0.19 ^c,d^	83.55 ± 0.47 ^e^	80.55 ± 0.15 ^f^
	a*	6.68 ± 0.24 ^a^	2.48 ± 0.05 ^b^	1.53 ± 0.05 ^c^	1.08 ± 0.01 ^d^	−1.68 ± 0.08 ^e^	−2.85 ± 0.11 ^f^	−4.82 ± 0.16 ^g^
	b*	8.56 ± 0.30 ^g^	10.12 ± 0.04 ^f^	11.13 ± 0.12 ^e^	13.29 ± 0.21 ^d^	17.90 ± 0.04 ^c^	19.85 ± 0.11 ^b^	30.95 ± 0.22 ^a^
	Δ*E*	16.42 ± 0.19 ^e^	15.57 ± 0.13 ^f^	13.23 ± 0.08 ^g^	17.11 ± 0.09 ^d^	21.16 ± 0.14 ^c^	23.40 ± 0.33 ^b^	34.66 ± 0.15 ^a^
CGR/ OBDs-5%	Color	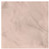	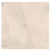	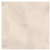	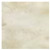	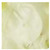	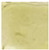	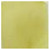
	L*	88.81 ± 0.31 ^a^	85.06 ± 0.48 ^c^	86.83 ± 0.45 ^b^	85.39 ± 0.15 ^c^	82.36 ± 0.02 ^d^	82.78 ± 0.13 ^d^	79.52 ± 0.37 ^e^
	a*	3.68 ± 0.17 ^a^	3.28 ± 0.11 ^b^	2.75 ± 0.12 ^c^	0.98 ± 0.08 ^d^	−3.04 ± 0.05 ^e^	−4.25 ± 0.09 ^f^	−5.43 ± 0.06 ^g^
	b*	6.87 ± 0.01 ^g^	9.38 ± 0.04 ^f^	10.40 ± 0.07 ^e^	12.68 ± 0.05 ^d^	18.43 ± 0.04 ^c^	21.74 ± 0.21 ^b^	27.04 ± 0.09 ^a^
	Δ*E*	15.51 ± 0.27 ^e^	13.41 ± 0.30 ^f^	10.70 ± 0.15 ^g^	16.49 ± 0.06 ^d^	22.72 ± 0.11 ^c^	25.80 ± 0.18 ^b^	31.09 ± 0.58 ^a^

Different superscript letters in the same line indicate statistically significant differences (*p* < 0.05).

## Data Availability

The original contributions presented in the study are included in the article/Supplementary Materials. Further inquiries can be directed to the corresponding author.

## Supplementary Materials

The following supporting information can be downloaded at: https://www.mdpi.com/article/10.3390/foods14193320/s1, Figure S1: Optical properties (A) images and (B) light transmittance of different composite films; Figure S2: (A) Hemolysis ratio and (B) OM values of different composite films. (C) Changes in visual appearance of films during soil burial period; Figure S3: The release rate of CEO from CGR/CEO-5% and CGR/OBDs-5% films in (A) 3% acetic acid, (B) 50% ethanol and (C) 95% ethanol. The release rate of OBDs from CGR/OBDs-5% film in (D) 3% acetic acid, (E) 50% ethanol and (F) 95% ethanol at different temperatures; Figure S4: Photos of the color change of the films under ultraviolet light irradiation; Figure S5: A correlation analysis between the incremental changes in the ΔE value of CG/ROSA and CGR/OBDs-5% films and the pH (A, D), TVB-N (B, E), and TVC (C, F) content of shrimps during the storage; Table S1: Surface color difference in different composite films. References [26,27,28,44] are cited in the Supplementary Materials.

## Abbreviations

The following abbreviations are used in this manuscript:

CEO	Citronella essential oil
ROSA	Red onion skins for the extraction of anthocyanins
β-CD	β-cyclodextrin
OBDs	Encapsulation of CEO within β-cyclodextrin
GEL	Gelatin
CAR	κ-carrageenan
CG	a κ-carrageenan/gelatin-based composite film
CGR	a κ-carrageenan/gelatin-based composite film incorporating ROSA
CGR/OBDs	a κ-carrageenan/gelatin-based composite film incorporating ROSA and OBDs
SEM	scanning electron microscopy
FTIR	Fourier-transform infrared spectroscopy
XRD	X-ray diffraction
*S. aureus*	*Staphylococcus aureus*
*E. coli*	*Escherichia coli*
