# Supplementary material for: Development of Intelligent and Active Gelatin-Based Packaging Film Incorporating Red Onion Anthocyanins and Encapsulated Citronella Oil
Source: Foods. 2025 Sep 25;14(19):3320. doi: 10.3390/foods14193320 (PMC12524074; doi:10.3390/foods14193320)
Supplement: Supplementary file 1 [file foods-14-03320-s001.zip › foods-3814469-supplementary material.pdf]

Development of Intelligent and Active Gelatin-based Packaging Film Incorporating Red Onion Anthocyanins and Encapsulated Citronella Oil

Zhaolan Yan [1], Kun Wang [1,*], Bingbing Xia [2], Jintao Wu [1] and Hongxu Chen[1]

1 Faculty of Food Science and Engineering, Kunming University of Science and Technology, Kunming 650500, China

2 Sorbonne Université, CNRS, Institut des NanoSciences de Paris (INSP), SAFIR, 75005

* Correspondence: 20210218@kust.edu.cn; Tel.: +86087165920293

1. Materials and methods

1.1. Extraction of red onion skins anthocyanins

Referring to the methodology described by Ali et al.[1], the extract solution comprised 75% ethanol, acidified with glacial acetic acid in a 6:1 ratio. Red onion skins were combined with the extract solution at a material-to-liquid ratio of 1:2. Initially, half of the extract solution was added, and the mixture was thoroughly stirred. The solution was then subjected to ultrasonic extraction in a water bath at 25 °C, maintaining a frequency of 40 kHz, for a duration of 20 to 30 minutes. Subsequently, the solution was allowed to stand for 1 hour at room temperature in the dark. The mixture was subsequently filtered using a filter bag to obtain the supernatant. The aforementioned steps were repeated with the remaining extraction liquid and filter residue, followed by a second filtration to collect additional supernatant. The combined extract was concentrated using a rotary evaporator to yield an anthocyanin concentrate, which was then freeze-dried to produce anthocyanin powder. For testing UV-vis spectroscopy of anthocyanins, 0.16 g of ROSA was dissolved in 100 mL of distilled water, resulting in an anthocyanin solution with an initial pH of 4. To adjust the pH of the solution within the range of 1.0 to 12.0, 1 mol/L HCl and 1 mol/L NaOH are utilized. The absorption spectrum of the anthocyanin solution was then scanned from 300 to 800 nm using a UV-visible spectrophotometer. Additionally, images of the anthocyanins' colors were captured at various pH levels.

1.2. Preparation and characterization of OBDs

The method employed for the preparation of OBD capsules was adapted with minor modifications from the protocol outlined by Chen et al. [2], the OBDs capsules were synthesized using a co-precipitation technique, maintaining a weight ratio of CEO to β-CD at 1:5. To create a saturated solution, β-CD was dissolved in deionized water at 60 °C with a concentration of 1:20 (w/v). Subsequently, the CEO solution was incrementally added to the saturated β-CD solution. This CEO solution was previously prepared by dissolving 1 g of CEO in 60% ethanol at a 1:5 (w/v) ratio, along with the addition of 0.25% (w/v) Tween 80. The resulting OBDs solution was subjected to continuous stirring for 3 hours before being cooled to room temperature. The mixture was stored at 4 °C overnight. Thereafter, the OBDs solution underwent centrifugation centrifuged at 8000 r/min for 15 minutes, followed by two washes with absolute ethanol, and was dried with hot air at 40 °C until a constant weight was achieved. Finally, the microcapsules were stored in a dry container for future use.

In accordance with the methodology detailed by Sun et al. [3], the antioxidant capabilities of citronella/β-cyclodextrin at varying concentrations (1%, 3%, 5%, and 7%) were assessed using DPPH and ABTS free radical scavenging assays. For the ABTS assay, 0.4 mL of the test solution was mixed with 2 mL of a 0.1 mM DPPH ethanol solution and incubated in the dark for 3 hours. The absorbance of the mixture was subsequently measured at 517 nm using a UV/visible spectrophotometer. In the ABTS assay, a stock solution was prepared by reacting 7.4 mM ABTS with 2.6 mM potassium persulfate under dark conditions for 16 hours. Subsequently, 0.5 mL of the sample solution was mixed with 5 mL of diluted $ABTS^+$ solution (with an absorbance of 0.70 ± 0.05) and allowed to react at room temperature for 2 hours. The absorbance was recorded at a wavelength of 734 nm. The free radical scavenging rates for DPPH and ABTS were determined using a specified formula:

$$\text{Radical scavenging rate (\%)} = (A_0 - A_f) / A_0 \times 100$$

In this formula, A_0 and A_f represent the absorbance of the free radical solution without and with the test solution, respectively.

An adapted method from Bansal et al. [4] was employed to assess the antibacterial activity of essential oil microcapsules. A bacterial suspension of 10^8 CFU/mL was evenly spread across an agar plate using a coating rod. Subsequently, an、a 10 mm diameter hole was created in the agar using a punch. Following this, the OBDs solution was introduced into the hole and incubated at 37 °C for 24 hours. Finally, the diameter of the inhibition zone was measured.

1.3. Preparation and characterization of composite films

1.3.1 Antibacterial activity and mechanism of films

Six film samples, each with dimensions of 2 cm × 2 cm, were subjected to UV irradiation for 20 minutes and then immersed in 5 mL of bacterial suspension (10^6 CFU/mL). The resulting mixture was incubated at 37 °C with shaking at 120 rpm, while a bacterial suspension devoid of the film served as the control. Following a 12-hour incubation period, 500 μL of the suspension was withdrawn and serially diluted using sterile physiological saline to achieve the desired gradient. A 100 μL aliquot from the diluted suspension was plated onto LB agar plates, which were subsequently incubated at 37 °C for 24 hours to facilitate colony analysis. The antibacterial rate was determined using a specific formula:

$$\text{Antibacterial rate (\%)} = (N_0 - N_n) / N_0 \times 100$$

In this context, N_0 and N_n represent the number of colonies (CFU/mL) without film treatment and after film treatment, respectively.

2. Results and discussion

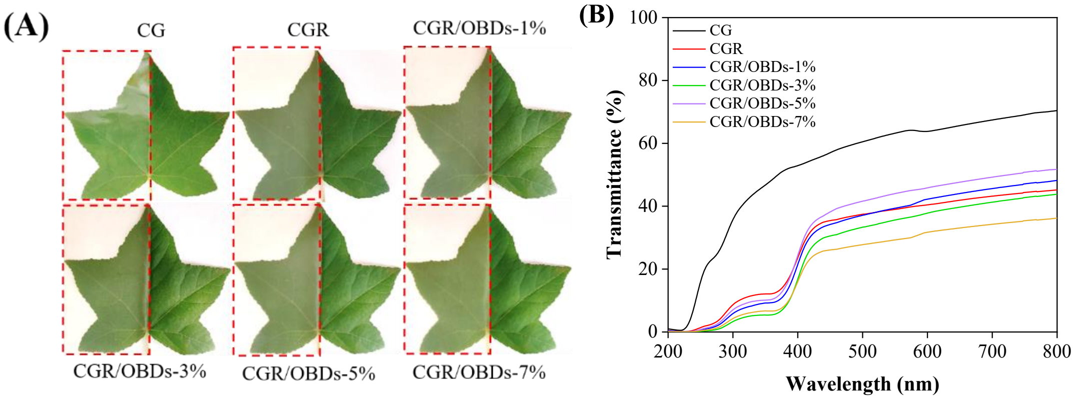

Figure S1. Optical properties (A) Imagesand (B) light transmittance of different composite films.

Table S1. Surface color difference of different composite films.

Film	L^*	a^*	b^*	ΔE
CG	96.16 ± 0.19^{a}	-1.07 ± 0.11^{d}	2.60 ± 0.05^{f}	1.72 ± 0.04^{e}
CG/ROSA	90.17 ± 0.34^{c}	1.49 ± 0.04^{a}	8.22 ± 0.08^{e}	9.74 ± 0.24^{d}
CGR/OBDs-1%	91.43 ± 0.20^{b}	1.40 ± 0.01^{ab}	10.21 ± 0.08^{d}	10.66 ± 0.11^{c}
CGR/OBDs-3%	89.92 ± 0.06^{cd}	1.36 ± 0.02^{b}	10.80 ± 0.11^{c}	11.91 ± 0.13^{b}
CGR/OBDs-5%	89.77 ± 0.09^{cd}	1.29 ± 0.02^{bc}	12.27 ± 0.05^{b}	13.21 ± 0.07^{a}
CGR/OBDs-7%	89.56 ± 0.08^{d}	1.21 ± 0.04^{c}	12.48 ± 0.11^{a}	13.48 ± 0.12^{a}

Different superscript letters in the same column indicate statistically significant differences ($p<0.05$).

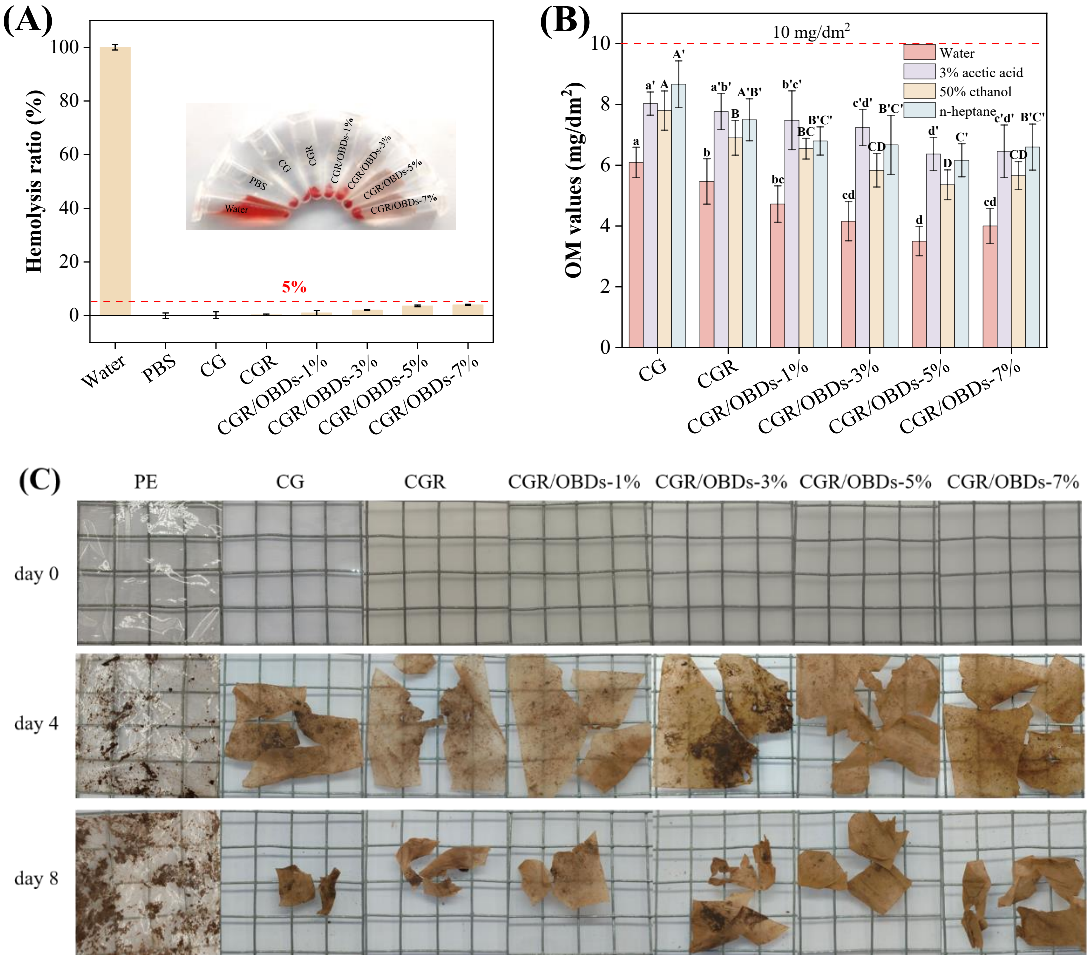

Figure S2. (A) Hemolysis ratio and (B) OM values of different composite films. (C) Changes in visual appearance of films during soil burial period.

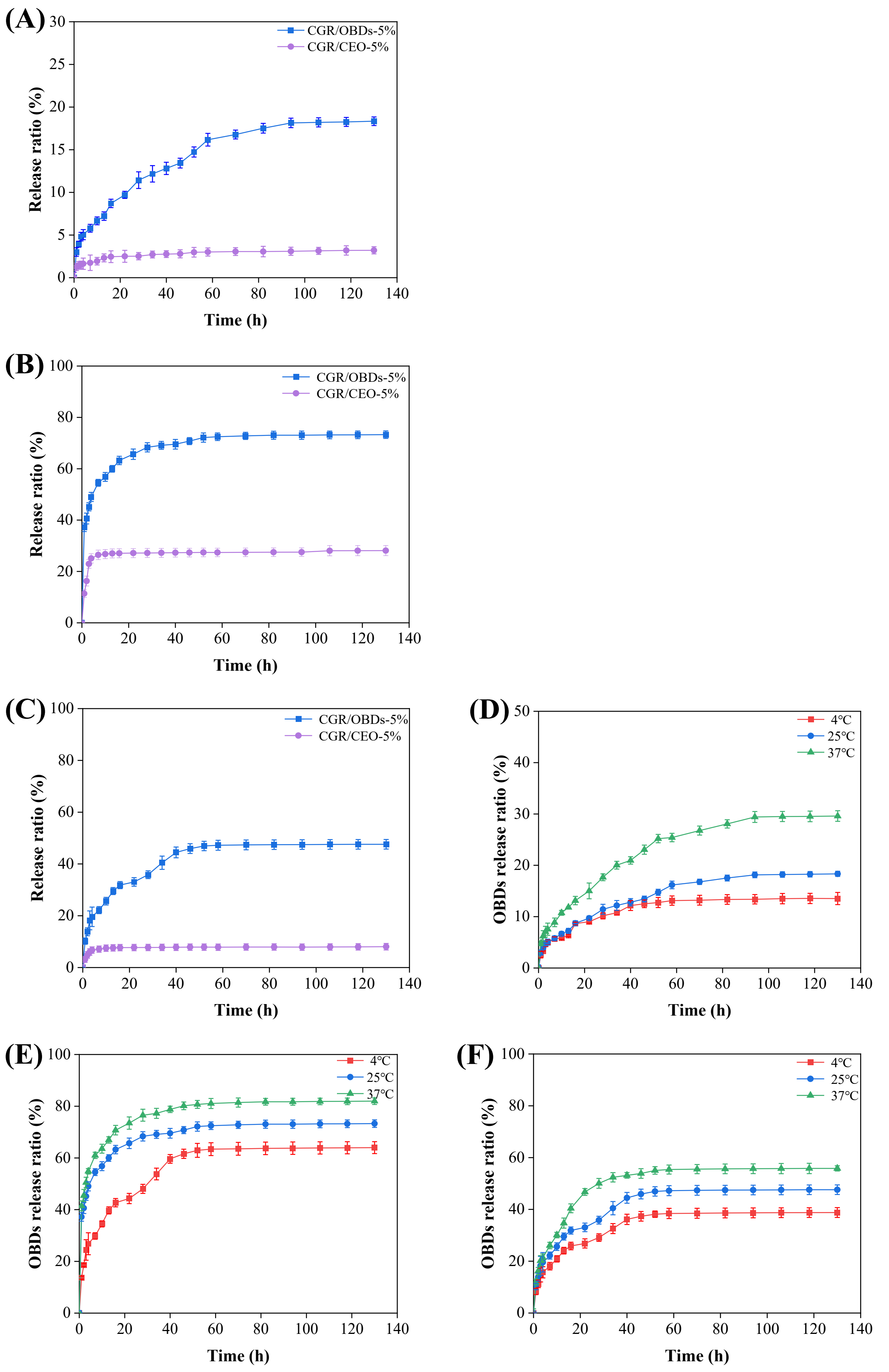

Figure S3. The release rate of CEO from CGR/CEO-5% and CGR/OBDs-5% films in (A) 3% acetic acid, (B) 50% ethanol and (C) 95% ethanol. The release rate of OBDs from CGR/OBDs-5%

film in (D) 3% acetic acid, (E) 50% ethanol and (F) 95% ethanol at different temperatures.

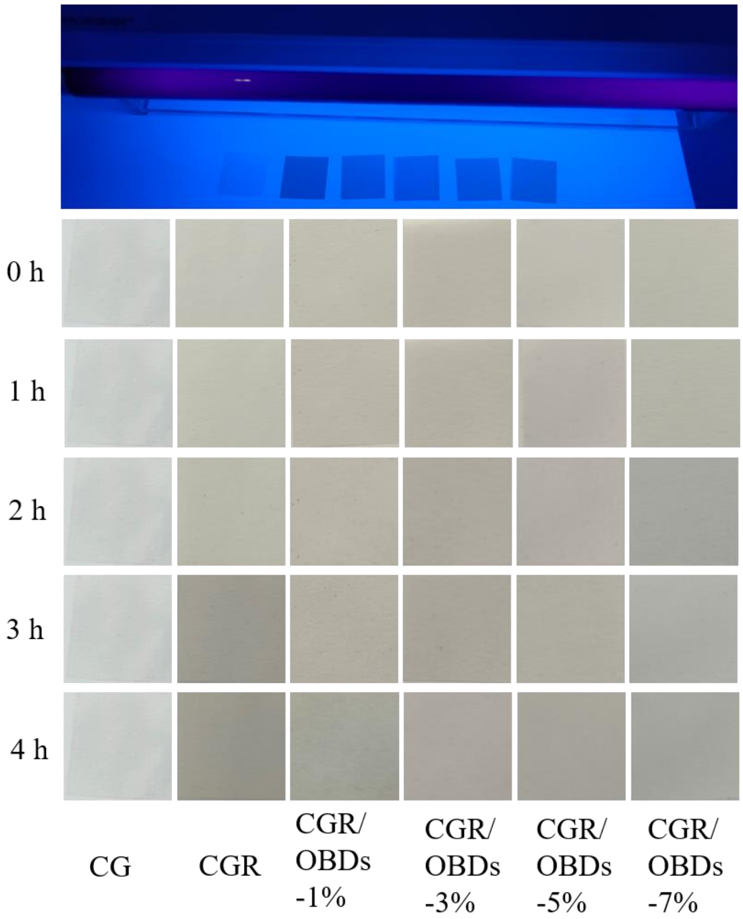

Figure S4. Photos of the color change of the films under ultraviolet light irradiation.

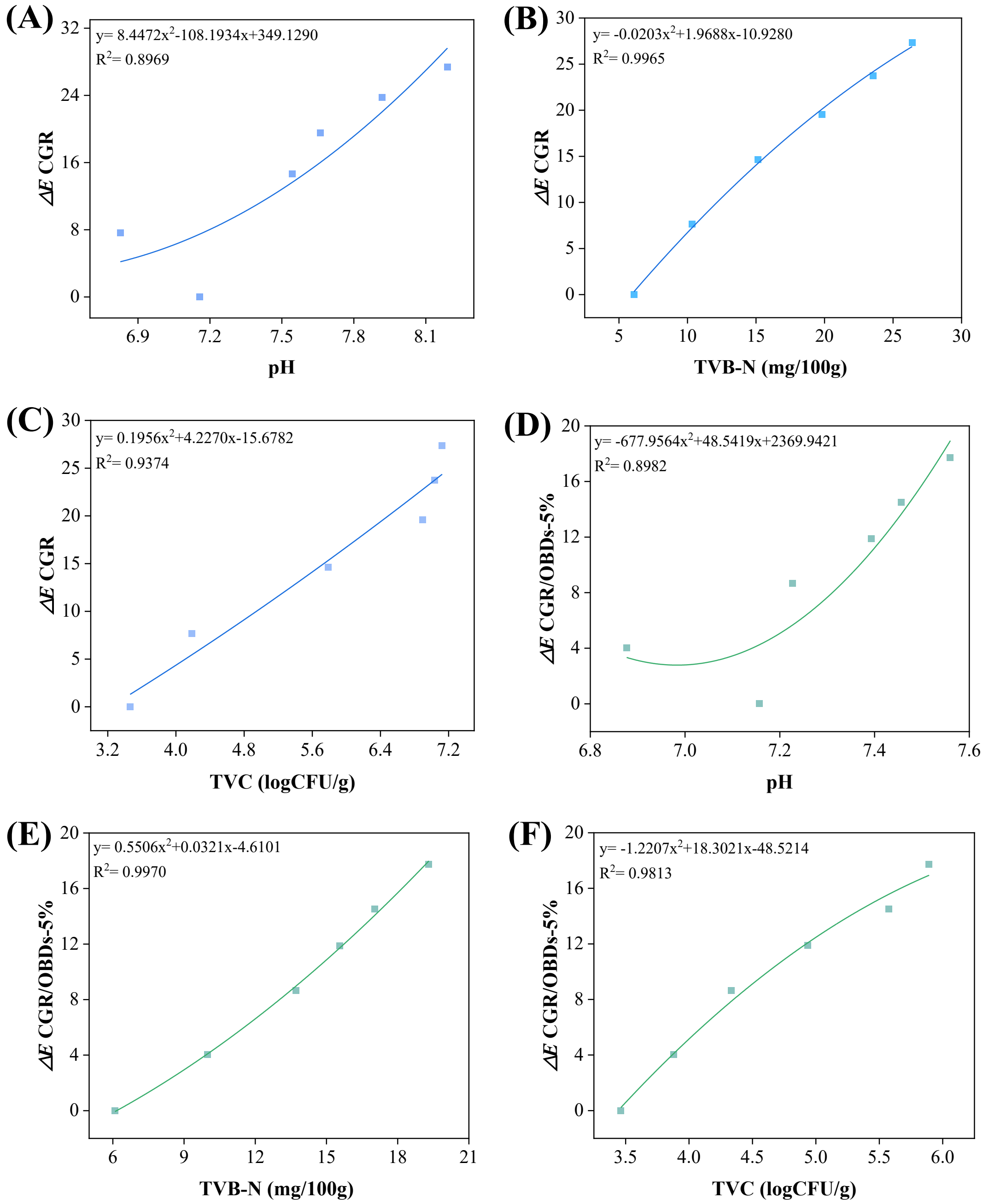

Figure S5. A correlation analysis between the incremental changes in the ΔE value of CG/ROSA and CGR/OBDs-5% films and the pH (A, D), TVB-N (B, E), and TVC (C, F) content of shrimps during the storage.

Reference

1. Ali, H.M.; Attia, M.H.; Rashed, E.N. Enhancing the Stability of Strawberry Anthocyanins Complexed to β-Cyclodextrin and Starch toward Heat, Oxidation, and Irradiation. ACS Omega 2024, 9, 5319-5329, doi:10.1021/acsomega.3c06311.
2. Chen, Z.; Zong, L.; Chen, C.; Xie, J. Development and characterization of PVA-Starch active films incorporated with β-cyclodextrin inclusion complex embedding lemongrass (Cymbopogon citratus) oil. Food Packaging and Shelf Life 2020, 26, 100565, doi:https://doi.org/10.1016/j.fpsl.2020.100565.
3. Sun, F.; Zhao, J.; Shan, P.; Wang, K.; Li, H.; Peng, L. Gelatin-based composite film integrated with nanocellulose and extract-metal complex derived from coffee leaf for sustainable and active food packaging. Food Hydrocolloids 2025, 159, 110610, doi:https://doi.org/10.1016/j.foodhyd.2024.110610.
4. Bansal, H.; Sharma, A.; Singh, S.; Mehta, S.K. Fabrication and application of buckwheat starch-based sustained-release composite films infused with curry leaf essential oil/β-cyclodextrin micro-capsules for preservation of green grapes. Food Hydrocolloids 2025, 159, 110666, doi:https://doi.org/10.1016/j.foodhyd.2024.110666.
